# The polygenic architecture of left ventricular mass mirrors the clinical epidemiology

**DOI:** 10.1038/s41598-020-64525-z

**Published:** 2020-05-05

**Authors:** Jonathan D. Mosley, Rebecca T. Levinson, Eric Farber-Eger, Todd L. Edwards, Jacklyn N. Hellwege, Adriana M. Hung, Ayush Giri, Megan M. Shuey, Christian M. Shaffer, Mingjian Shi, Evan L. Brittain, Wendy K. Chung, Iftikhar J. Kullo, Adelaide M. Arruda-Olson, Gail P. Jarvik, Eric B. Larson, David R. Crosslin, Marc S. Williams, Ken M. Borthwick, Hakon Hakonarson, Joshua C. Denny, Thomas J. Wang, Charles M. Stein, Dan M. Roden, Quinn S. Wells

**Affiliations:** 1 Department of Medicine, Vanderbilt University Medical Center, Nashville, TN USA; 2 Department of Biomedical Informatics, Vanderbilt University Medical Center, Nashville, TN USA; 3 Vanderbilt Institute for Clinical and Translational Research, Vanderbilt University Medical Center, Nashville, TN USA; 4 Vanderbilt Epidemiology Center, Vanderbilt University Medical Center, Nashville, TN USA; 5 Tennessee Valley Healthcare System (626), Vanderbilt University, Nashville, TN USA; 6 Department of Obstetrics and Gynecology, Vanderbilt University Medical Center, Nashville, TN USA; 7 Institute for Medicine and Public Health, Vanderbilt University Medical Center, Nashville, TN USA; 8 Office of Research & Development, Department of Veterans Affairs, Washington DC, DC USA; 9 Departments of Pediatrics and Medicine, Columbia University Medical Center, New York, NY USA; 10 Department of Cardiovascular Diseases, Mayo Clinic, Rochester, MN USA; 11 Departments of Medicine (Medical Genetics) and Genome Sciences, University of Washington, Seattle, WA USA; 12 Kaiser Permanente Washington Health Research Institute and Department of Medicine, University of Washington, Seattle, WA USA; 13 Departments of Biomedical Informatics and Medical Education, University of Washington, Seattle, WA USA; 14 Genomic Medicine Institute, Geisinger, Danville, PA USA; 15 Biomedical and Translational Informatics, Geisinger, Danville, PA USA; 16 Center for Applied Genomics, Division of Human Genetics, Department of Pediatrics, The Children’s Hospital of Philadelphia, University of Pennsylvania, Philadelphia, PA USA; 17 Department of Pharmacology, Vanderbilt University Medical Center, Nashville, TN USA

**Keywords:** Cardiac hypertrophy, Genetic association study

## Abstract

Left ventricular (LV) mass is a prognostic biomarker for incident heart disease and all-cause mortality. Large-scale genome-wide association studies have identified few SNPs associated with LV mass. We hypothesized that a polygenic discovery approach using LV mass measurements made in a clinical population would identify risk factors and diseases associated with adverse LV remodeling. We developed a polygenic single nucleotide polymorphism-based predictor of LV mass in 7,601 individuals with LV mass measurements made during routine clinical care. We tested for associations between this predictor and 894 clinical diagnoses measured in 58,838 unrelated genotyped individuals. There were 29 clinical phenotypes associated with the LV mass genetic predictor at FDR q < 0.05. Genetically predicted higher LV mass was associated with modifiable cardiac risk factors, diagnoses related to organ dysfunction and conditions associated with abnormal cardiac structure including heart failure and atrial fibrillation. Secondary analyses using polygenic predictors confirmed a significant association between higher LV mass and body mass index and, in men, associations with coronary atherosclerosis and systolic blood pressure. In summary, these analyses show that LV mass-associated genetic variability associates with diagnoses of cardiac diseases and with modifiable risk factors which contribute to these diseases.

## Introduction

Left ventricular (LV) mass captures the cardiac response to the cumulative exposure of a diverse set of risk factors, and is strongly predictive of future cardiac events and all-cause mortality^1–3^. Determining the genetic factors associated with LV mass could identify important predisposing mechanisms that contribute to heart disease. Three genome-wide association studies (GWAS) have only identified a single nucleotide polymorphism (SNP) associated with LV mass^4–6^. Thus, the genetic architecture underlying LV mass remains uncharacterized.

An individual’s LV mass can change over time (referred to as LV remodeling). For instance, LV mass may increase due to prolonged exposure to risk factors such as high blood pressure, metabolic abnormalities and cardiac diseases^7^. In contrast, treating these risk factors using medications or other interventions attenuates or reverses these LV mass increases over time. These changes suggest that important processes contributing to adverse LV remodeling have extended latencies and are modulated by environmental influences.

We hypothesized that defining the polygenic architecture of LV mass in a clinical population comprised of healthy and diseased individuals, would identify genetic variation associated with variability in LV mass within that population. To test this hypothesis, we developed a SNP-based polygenic predictor of LV mass in a genotyped population of individuals who received transthoracic echocardiography (TTE) as part of routine clinical care at the Vanderbilt University Medical Center (VUMC). To identify the genetic diseases associated with this predictor, we interrogated it against a large collection of diseases ascertained through the Electronic Medical Records and Genomics (eMERGE) network, a consortium of medical centers with EHR-linked DNA biobanks^8,9^. We show that genetic diagnoses associated with LV mass include modifiable risk factors that suggest targets for directed treatment and prevention efforts within the clinical population.

## Results

### TTE population

The TTE population was 53% male and had a mean age of 64 (standard deviation[s.d.], 12) years (Supplementary Table 1). The most prevalent diagnoses were hypertension (81%), respiratory symptoms (75%), arrhythmias (68%) and lipid disorders (67%). The mean LV mass was 212 (s.d. 72) grams in men and 37.0% had an LV mass greater than the upper limit of normal in men (224 grams). Among women, mean LV mass was 155 (s.d. 54) grams and 37.8% had a mass over 162 grams.

### GWAS analysis

We performed a GWAS to determine whether there were common SNP variants associated with LV mass. There were no SNPs associated with LV mass at the genome-wide significant threshold of p < 5 × 10^-8^ (Supplementary Figure 1). A prior GWAS reported an association between LV mass and the SNP rs2255167-T, located within the *TTN* gene^6^. There was a similar direction of effect for this SNP in these analyses, but the association was not significant (β = 0.012, standard error = 0.007, p = 0.06).

### PheWAS analysis

An alternative unbiased discovery strategy to identify genetic associations is to construct a polygenic predictor comprising common SNPs associated with the phenotype. We validated this predictor against the PheWAS diagnosis of cardiomegaly, which is a clinical diagnosis of an enlarged heart, and corresponds to an elevated LV mass. Within the training data set used to build the polygenic predictor, both measured LV mass and the genetic predictor were strongly positive associated (p < 2 × 10^-16^) with the risk of a cardiomegaly diagnosis (Table 1). While adjusting for measured LV mass eliminated the association between cardiomegaly and the predictor (p = 0.06), adjusting for either body mass index or height only minimally attenuated the association (p < 2 × 10^-16^). Thus, the phenotypic variation captured by the genetic predictor corresponds to LV mass. A genetic predictor derived from permuted LV mass measurements was not associated with the phenotype, indicating that a genetic predictor from a random phenotype does not associate with the cardiomegaly diagnosis. In two independent validation sets that did not include individuals used to build the genetic predictor, the LV mass genetic predictor was significantly positively associated with cardiomegaly (Table 1). Thus, the genetic predictor demonstrated the expected associations with the cardiomegaly positive control phenotype.

**Table 1:** Validation of the LV mass polygenic predictor (PRS).

Data source	Independent variable	Additional covariate^a^	OR (95% CI)^b^	P-value
Derivation set^c^	LV Mass measured	None	2.1 (2.0–2.3)	<2 × 10^−16^
	LV Mass PRS	None	2.0 (1.9–2.1)	<2 × 10^−16^
	LV Mass PRS	LV Mass measured	1.1 (0.99–1.31)	0.06
	LV Mass PRS	Height	2.0 (1.9–2.2)	<2 × 10^−16^
	LV Mass PRS	BMI	1.9 (1.8–2.1)	<2 × 10^−16^
	LV Mass permuted PRS	None	0.99 (0.94–1.04)	0.66
Validation set 1: BioVU^d^	LV Mass PRS	None	1.08 (1.02–1.13)	0.002
Validation set 2: eMERGE^e^	LV Mass PRS	None	1.10 (1.06–1.14)	4.1 × 10^−7^

^a^Additional covariate added to the logistic regression model.

^b^Analyses are based on a logistic regression model using the PheWAS phenotype cardiomegaly as the dependent variable and specified independent variable. All independent variables were set to have a standard deviation of 1. All models were additionally adjusted for age, sex and 5 principal components.

^c^The data set used to develop the PRS. There were 2929 cases and 3416 controls for cardiomegaly.

^d^Cases (n = 2,624) and controls (n = 16,682) for the cardiomegaly phenotype that were not part of the derivation set.

^e^Cases (n = 5,982) and controls (n = 21,198) for the cardiomegaly phenotype that were not part of the derivation set.

**Figure 1:**
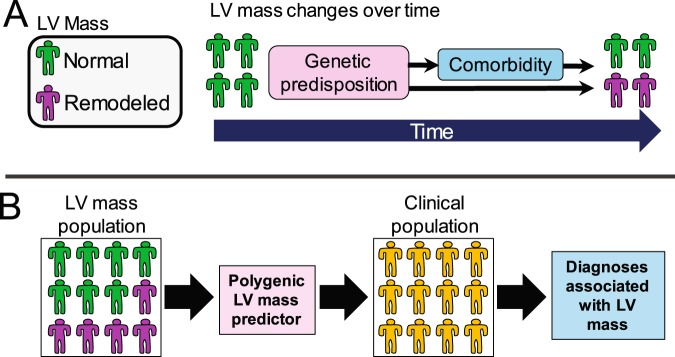
Overview of rationale and approach. (**A**) Over time, some individuals undergo changes in LV mass (remodeling). Among factors contributing to these changes is a genetic predisposition which includes disease variants which directly affect cardiac structure and variants which predispose to comorbidities which secondarily lead to remodeling. (**B**) LV mass measurements were derived from a clinical population who had undergone transthoracic echocardiography (TTE). LV mass variation in this population was modeled using a SNP-based polygenic predictor. This predictor was then tested for associations with clinical phenotypes ascertained in a larger clinical population. Clinical diagnoses associated with the predictor are associated with genetic factors that also associate with LV mass variation in the TTE population.

**Figure 2:**
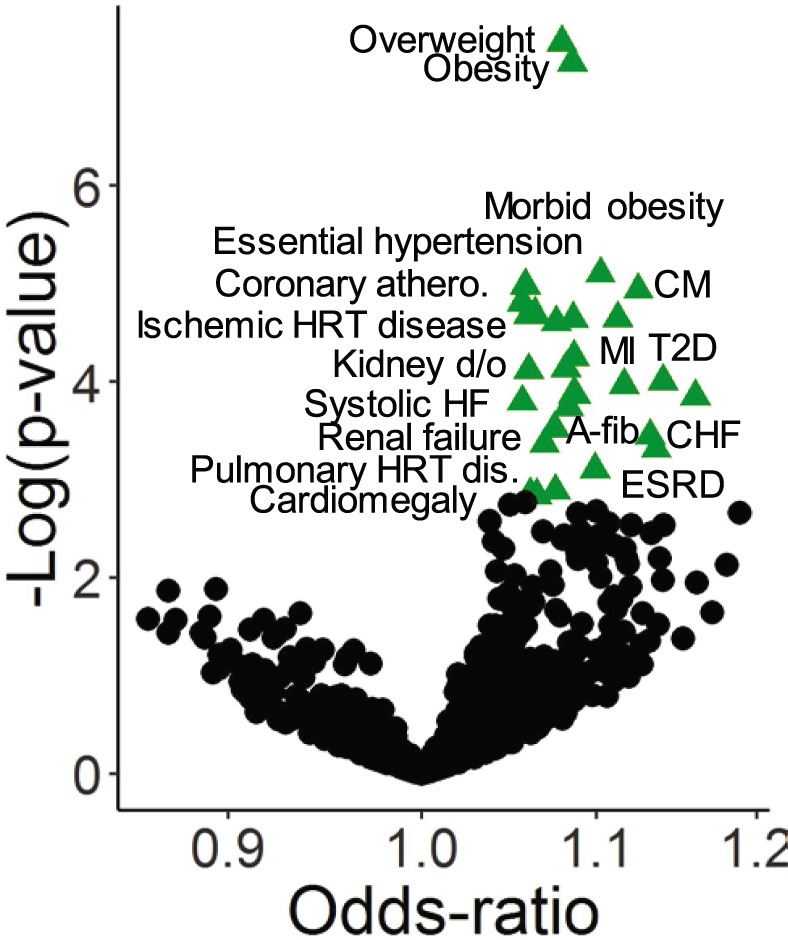
Diagnoses associated with genetically predicted LV mass. The funnel plot summarizes clinical associations between a PheWAS phenotype and predicted LV Mass. Each point represents a logistic regression association analysis, adjusting for age, sex and 5 principal components. Odds-ratios are the risk associated with a 1 standard deviation change in the value of the LV mass genetic predictor. Points highlighted by green triangles are significant at a false discover rate (FDR) q < 0.05, and selected points are labelled.

We used this predictor to perform a phenome scan to identify clinical diagnosis associated with genetic variability that also associates with LV mass (Fig. 1 and Supplementary Table 2). The estimated proportion of the LV mass variance accounted for by the SNPs used to construct the predictor was 12.4%. There were 29 diagnoses associated with the genetic predictor (FDR q < 0.05) (Fig. 2 and Supplementary Table 3). All significant associations had positive odds-ratios, indicating that higher genetically predicted LV mass was associated with an increased risk of the clinical phenotype. Among the significant associations were modifiable risk factors including obesity (p = 5.6 × 10^-8^), hypertension (p = 1.0 × 10^-5^), coronary artery disease (p = 2.0 × 10^-5^), and type 2 diabetes (T2D) (p = 7.6 × 10^-5^) as well as cardiac diagnoses including cardiomyopathies (p = 1.2 × 10^-5^), cardiomegaly (p = 1.8 × 10^-4^), and atrial fibrillation/flutter (p = 4.3 × 10^-4^). There were also associations with renal and pulmonary vascular disease phenotypes.

### Genetic risk score analyses

Four of the associations were with modifiable risk factors that represent potential targets for intervention. To verify these associations, we employed a Mendelian Randomization approach to determine whether genetic risk scores (GRSs) derived from large GWAS studies for these risk factors associated with LV mass in the TTE population. A GRSs for each risk factor was strongly associated with a positive control phenotype measured in an independent population (Supplementary Table 4). There was a consistent positive association between a BMI GRS and LV Mass in both genders (Table 2). Among men, there were also significant positive associations with systolic blood pressure (SBP) (p = 0.03) and coronary artery disease (p = 0.03) with consistent directions of effects across all association methods evaluated, though the point estimates for the effect estimates were smaller for SBP when using the MR-Egger (change in log LV mass per unit change in SBP = 0.002) and Weighted Median (0.003) methods, which are less sensitive to the effects of pleiotropy, as compared to the Inverse-variance weighted average meta-analysis method (IVWM) (0.004).

**Table 2:** Associations between genetic predictors for selected risk factors and LV mass, by sex.

Risk factor	Males	P-value	Females	P-value
	Change in LV Mass^a^		Change in LV Mass^a^	
**Inverse-variance weighted average meta-analysis**				
Body mass index	**0.079 (0.015)**	**1.2 × 10**^**−7**^	**0.147 (0.016)**	**1.7 × 10**^**−20**^
Systolic blood pressure	**0.004 (0.002)**	**0.03**	0.001 (0.001)	0.29
Coronary artery disease	**0.027 (0.013)**	**0.03**	−0.002 (0.014)	0.87
Type 2 diabetes (T2D)	−0.001 (0.008)	0.92^b^	**0.018 (0.008)**	**0.02**
T2D, excluding BMI SNPs^d^	0.017 (0.015)	0.25	**−0.008 (0.015)**	**0.62**
**MR-Egger method**				
Body mass index	**0.146 (0.047)**	**0.002**	**0.168 (0.050)**	**0.001**
Systolic blood pressure	0.002 (0.007)	0.7	0.000 (0.007)	0.95
Coronary artery disease	0.044 (0.030)	0.15	−0.040 (0.033)	0.23
Type 2 diabetes (T2D)	−0.042 (0.018)	0.02^c^	0.004 (0.019)	0.82
T2D, excluding BMI SNPs^d^	−0.026 (0.046)	0.58	0.049 (0.049)	0.31
**Weighted median method**				
Body mass index	**0.114 (0.025)**	**4.0 × 10**^**−6**^	**0.145 (0.029)**	**6.8 × 10**^**−7**^
Systolic blood pressure	0.003 (0.003)	0.24	−0.002 (0.003)	0.51
Coronary artery disease	0.037 (0.020)	0.06	−0.020 (0.022)	0.37
Type 2 diabetes (T2D)	−0.017 (0.014)	0.23	0.008 (0.015)	0.6
T2D, excluding BMI SNPs^d^	0.006 (0.023)	0.81	−0.003 (0.025)	0.9

^a^Estimates (standard error) represent the changes in the log(LV mass) per unit change of the risk factor.

^b^Heterogeneity p < 0.05.

^c^MR-Egger intercept p < 0.05.

^d^Excludes SNPs associated with BMI at p < 0.05.

Among women, increased type 2 diabetes (T2D) genetic risk was associated with higher LV mass (Table 2). However, the effect estimates were considerably weaker when using the MR-Egger and Weighted Median methods, suggesting pleiotropy. Also, among males, there was evidence of heterogeneity among the SNP associations (heterogeneity p-value = 0.02 and the MR-Egger intercept p-value = 0.01). Elevated BMI is a risk factor of T2D and when the analyses were repeated after excluding BMI-associated SNPs from the GRS, the T2D association was not significant in either men or women, suggesting the T2D association was likely due to the effects of elevated BMI on both T2D risk and LV mass. In sum, these analyses confirm the observed associations between LV Mass and obesity and, in males, associations with both hypertension and coronary artery disease.

## Discussion

We used TTE measurements taken in a heterogeneous clinical population to identify diseases and risk factors associated with LV mass variability. We identified 29 clinical diagnoses associated with genetic variation that also associates with LV mass. We used GRSs to confirm associations between genetic predictors of adiposity, blood pressure and atherosclerotic disease, and LV mass, and found that a genetic predisposition toward these risk factors associates with higher LV mass measurements. In aggregate, these analyses are in agreement with the known clinical epidemiology of LV remodeling, and extend our understanding of the genetic epidemiology of this phenotype.

An individual’s LV mass is not static and may increase over time due to the unmitigated effects of disease processes driven by gene-by-environment interactions. Thus, LV mass is a biomarker that measures the severity and duration of exposure to a broad range of pathological influences. Within a population, LV mass has been shown to be a prognostic measure of cardiac health^1–3^. Thus, the genetic architecture of LV mass in a clinical population could be expected to capture important genetic influences of disease.

The genetic architecture of LV mass has remained largely elusive to SNP-based discovery approaches, and only one significant SNP association has been reported^4–6^. We did not identify individual SNPs associated with LV mass, and we did not replicate the previously reported association in the *TTN* gene. To gain further insights into the genetic architecture, we leveraged a polygenic approach whereby we constructed a genetic risk score that captured the additive contributions of a large number of SNPs. We then employed PheWAS to identify clinical phenotypes associated with the genetic variation captured by this predictor^9^. Among the phenotypes associated with the GRS were diagnoses related to elevations in risk factors such as obesity and hypertension, diagnoses of heart disease including diagnoses of end-stage disease such as heart failure as well cardiac diseases such as coronary artery disease.

A prior study in a Japanese population used genetic correlation analyses, another polygenic approach, to test for associations between LV mass and 30 candidate diseases and reported significant associations with T2D, stroke risk and atrial fibrillation risk^5^. Our study, which employed a discovery-based approach, refined and extends these findings by identifying a much broader range of clinical phenotypes associated with LV mass. Indeed, the extended range of pathologies associated with LV mass demonstrate that genetic variation underlying variability in LV mass also underlies diagnoses affecting multiple organ systems including the kidney, lungs and heart, which may account for the strong association between elevated LV mass and mortality^3^.

Our findings have direct translational relevance to the target population from which the LV mass genetic predictor was derived. In the two sample approach used in these analyses, an association is observed when genetic variation associated with LV mass also associates with the disease or risk factor. The associations with obesity, hypertension and CAD indicate that genetic variation associated with increased risk for these diagnoses may also contribute to structural heart changes in our clinic population, an observation consistent with the Mendelian randomization analyses. It is important to note that the genetic architecture of a trait reflects gene-by-environmental interactions present in a population. These interactions can be exploited to develop approaches to attenuate genetic risk. For instance, the penetrance of obesity-associated genetic variation is modulated by environment^10,11^. Thus, one direct implication of our findings is that prevention efforts in the TTE population should be directed toward approaches with mitigate the genetic predisposition towards these risk factors including promoting health behaviors that reduce both obesity and CAD risk, and treating hypertension.

There were differences in the patterns of associations between men and women with respect to SBP and CAD. One explanation for the CAD difference is the fact that men at elevated CAD genetic risk are more likely to have CAD events, as compared to women with comparable genetic risk^12,13^. Thus, a CAD GRS is a poorer surrogate for CAD events among women, and this would lead to an attenuation of the association among women. For SBP, one reason that an association may not have been observed in women is that, for a given change in SBP, men manifest larger differences in LV mass^14^. Thus, the effect sizes in women may be beyond the power of this study to detect. An alternative explanation is that the women’s blood pressures may be under better control than men and, thus, they have not undergone remodeling to the extent present among men.

There are limitations to these analyses. We used clinical phenotypes defined using diagnostic codes primarily used for reimbursement and which may be under-ascertained or inaccurate, and which can lead to false negative associations. Furthermore, the set of diagnoses that we interrogated were not inclusive of all clinical diagnoses. The TTE data set was a real-world clinical data set and the clinical protocols used may have varied over time. However, despite this potential heterogeneity, we were still able to recapitulate known LV mass associations. We did not confirm that the LV mass genetic predictor associated with measured LV mass in an independent genotyped sample. Thus, it is possible, though unlikely, that the predictor is measuring genetic variation that is unrelated to LV mass. These analyses were limited to individuals of European ancestry, and future analyses in other ancestries are needed to describe the local epidemiology of these populations. Treatments and other environmental factors may cause LV mass reverse remodeling which will attenuate or eliminate associations between LV mass and the mitigated diagnoses or risk factors. Finally, these findings are most relevant to the population from which the TTE measures were derived. We view this as a strength, as this approach identifies potentially untreated genetic risk mechanisms that directly impact the population that the biomarker was measured in.

In summary, we leveraged the polygenic architecture underlying LV mass variability in a clinical population to identify clinical diagnoses associated with structural heart disease. Consistent with the prognostic nature of this phenotype, a genetic predictor of LV mass was associated with end-stage organ disease such as heart failure and kidney failure. Importantly, we also identified and confirmed associations with modifiable risk factors including obesity, hypertension and coronary artery disease. These findings highlight the power of polygenic methods to elucidate the genetic architecture of disease, as compared to SNP-based analyses, and extend our understanding of genetic modulators of LV remodeling in a clinical population. Importantly, our results suggest that well-recognized modifiable risk factors of LV remodeling associate with LV mass increases, suggesting that they are incompletely treated among the patients we studied. Future studies should assess whether genetic risk factor associations are similar in diverse populations.

## Methods

### Study populations

The echocardiography population was derived from the VUMC BioVU resource, a collection of individuals seen at VUMC whose EHR data was de-identified and linked to a DNA biobank constructed from discarded blood samples^15^. This IRB-approved resource includes individual-level clinical data and procedural reports (e.g., echocardiography). TTE measurements were extracted from VUMC’s clinical echocardiography database for adults over 35 years old who had TTEs performed between 2008 and 2016 and who had DNA available for SNP genotyping. The majority of subjects were identified as white; thus, analyses were restricted to individuals of genetic European ancestry (EA)^16^. The final echocardiography population comprised 7,601 unrelated individuals (Supplementary Table 1).

The phenome-wide association study (PheWAS) included EA individuals born prior to 1990 from the eMERGE network (phases 1-3) (n = 31,773, excluding VUMC)^8^ and additional BioVU subjects over 18 years old (n = 27,065) (Supplementary Table 2). The participating eMERGE sites were Columbia University, Geisinger, Marshfield Clinic, Northwestern University, Mayo Clinic, Harvard University, Mt. Sinai Health System, and Kaiser Permanente/University of Washington, Seattle.

Analyses were approved by each eMERGE institution’s Institutional Review Board (IRB)^8,15^.

### Genetic data

BioVU subjects underwent SNP genotyping using the Illumina Infinium Multi-Ethnic Genotyping Array (MEGA^EX^) platform. eMERGE subjects were genotyped on multiple platforms and underwent QC analyses and imputation as previously described^17,18^. Quality control (QC) analyses used PLINK v 1.90β3^19^ and included reconciling strand flips, verifying that allele frequencies were concordant among data sets, and identifying duplicate and related individuals (one of each pair of subjects with a pi-hat >0.05 was excluded)^17,20^. Data sets were standardized using the HRC-1000G-check tool v4.2.5 (http://www.well.ox.ac.uk/~wrayner/tools/) and pre-phased using SHAPEIT^21^. For the subjects with TTEs, data were imputed using IMPUTE2^22^ in conjunction with the 10/2014 release of the 1000 Genomes cosmopolitan reference haplotypes. All other genetic data for were imputed using the Michigan Imputation Server (HRC v1.1)^23^. Imputed data were filtered for a sample missingness rate <2%, a SNP missingness rate <4% and a SNP deviation from Hardy-Weinberg p < 10^-6^. There were 5,455,089 imputed SNPs with MAF > 1% that passed QC in all data sets. The LV mass genetic predictor was constructed using a LD-reduced (r-square<0.9) subset of 1,005,032 SNPs. Principal components were generated using the SNPRelate package^24^.

### Echocardiographic and phenotype data

LV mass was calculated using clinically acquired echocardiographic parameters according to the formula:^25^

$${\rm{LV}}\,{\rm{mass}}=0.8\{1.04[({[{\rm{LVEDd}}+{\rm{IVSd}}+{\rm{PWd}}]}^{3}\,-\,{{\rm{LVEDd}}}^{3})]\}\,+\,0.6$$

where LVEDd = LV internal diameter at end diastole; IVSd = interventricular septal thickness at end diastole; and PWd = LV posterior wall thickness at end diastole. There were 7,601 individuals with LV mass measurements with a value between 50 and 500 grams (g). For individuals with multiple TTEs, only measurements from the first were used. LV mass was log-transformed for these analyses. While LV mass is often indexed to body surface area or height, we used unindexed values to avoid spurious genetic associations caused by adjusting a phenotype by another highly heritable phenotype (referred to as collider bias)^26^.

PheWAS were conducted in the eMERGE and BioVU populations using clinical phecode phenotypes (https://phewas.mc.vanderbilt.edu/), which are collections of related ICD-9-CM (International Classification of Disease, Ninth revision) diagnosis codes^27,28^. Cases were individuals with two or more instances of a PheWAS diagnosis appearing in their medical record^29^. Phenotypes that affected a single sex (such as prostate cancer or uterine prolapse) were excluded. There were 894 clinical phenotypes with ≥300 cases (our minimum criteria for inclusion). Controls were subjects without the clinical phenotype or any closely related PheWAS code (using the standard phecode control groupings) and whose age (BioVU) or decade of birth (eMERGE) fell within the range of values observed among cases. The cardiomegaly PheWAS code (code 416) was used a positive control phenotype to validate the LV mass polygenic predictor. For these analyses, cases were individuals who had one or more instances of this diagnosis in their medical record, which is a more sensitive case definition.

### GWAS summary statistics

To further explore the relationship between LV mass and candidate phenotype associations, summary statistics from prior large-scale GWAS were used to construct genetic risk scores representing these candidate phenotypes. Specifically, summary statistics were obtained for coronary artery disease (CAD) from the CARDIOGRAM C4D consortium GWAS^30^, body mass index (BMI) from the GIANT Consortium^31,32^, systolic blood pressure (SBP) from a GWAS from the Million Veterans Program^33^, and type 2 diabetes (T2D) from the DIAGRAM Consortium^34^. Summary statistics were downloaded from the consortia websites.

### Analysis

GWAS was performed assuming an additive model and employed a multivariable linear model adjusted for age, sex and 10 principal components. A p < 5x10^-8^ was considered significant.

The vast majority of the heritability attributable to common SNPs is accounted for by SNPs that typically do not meet the criteria for genome-wide significance^35,36^. Thus, we used a modelling approach that assigns SNP weightings based on large numbers of common SNPs to construct a genetic risk score for LV mass. Genetically predicted LV mass was computed using a two-step approach, as previously described^9,16^. First, predictive weightings were assigned to SNPs using Bayesian sparse linear mixed modelling (BSLMM), as implemented in the GEMMA v0.95α package^37^. The BSLMM approach jointly models the contribution of all SNPs to the observed phenotypic variance by employing a hybrid of generalized linear mixed modelling and sparse regression models^38^. The models were adjusted for age on the date of the TTE, sex and 5 PCs; 100,000 sampling steps were run. The estimated proportion of additive genetic variance explained by the common SNPs used to model the SNP weightings is the median estimated value taken from the last 50,000 sampling steps^39^. SNP weightings are comprised of a small polygenic effect (α), a large effect (β) and a posterior probability that the SNP is in the large effect group (γ). The SNP weight (w) is computed from these estimates using the equation: w=α + βγ. The SNP weightings were used to compute a predicted LV mass for each individual in the PheWAS analysis (i.e. subjects without a TTE measurement) using the following equation

1 $${\rm{Predicted}}\,{\rm{feature}}\,{\rm{value}}=\mathop{\sum }\limits_{i=1}^{\#{\rm{SNPs}}}({{\rm{w}}}_{{\rm{i}}}\times {[{\rm{SNP}}{\rm{genotype}}]}_{{\rm{i}}})$$

where genotype is the number of alleles present for the SNP (coded as 0, 1 or 2).

To identify clinical phenotypes associated with genetically predicted LV mass, multivariable logistic regression, adjusting for 5 PCs, sex and either [birth decade and site for eMERGE sites] or [maximum age for BioVU], was used to test for an association with each PheWAS phenotype (dependent variable) and the predicted feature (independent variable) using the R PheWAS package^40^. Odds-ratios (ORs) are the risk of disease per standard deviation (s.d.) increase in the genetically predicted biomarker value. PheWAS analyses were run separately for the BioVU and eMERGE subjects and results were meta-analyzed using the METAL package^41^. To adjust for multiple testing, we employed a Benjamini-Hochberg (B-H) false discovery rate (FDR)^42^ adjustment and a q-value < 0.05, which has previously been shown to perform well for these analyses^9^, was considered significant.

To further characterize the candidate associations between the genetic risk score for LV mass and the PheWAS phenotypes, we generated weighted genetic risk scores for four candidate phenotypes and assessed their associations with LV mass within the TTE population. The genetic risk scores were based on summary statistics from GWAS of each phenotype. The SNPs comprising each GRS were selected using a clumping algorithm that identified an LD-reduced set (r-square < 0.05) of the most significantly-associated SNPs that had a minor allele frequency (MAF) > 5% and an association p-value < 5 × 10^-6^ in the original GWAS^43^. To validate the relevance of each GRS, its association was tested against the corresponding phenotype ascertained in BioVU subjects (n = 13,077). For continuous phenotypes (BMI and SBP), the phenotype represents the median for all available values for an individual. Binary phenotypes (coronary artery disease and type 2 diabetes) are based on PheWAS phenotypes.

Associations were tested using an inverse-variance weighted average meta-analysis (IVWA). Heterogeneity p-values are based on the Cochran’s Q statistic, and a low p-value indicates that that one or more variants in the GRS may be pleiotropic. Though less powered than the IVWA, associations were also tested by the MR-Egger and Weighted Median methods, which provide more accurate estimates of effect sizes in the presence of horizontal pleiotropy, strong outliers or invalid instrumental variables. Associations were measured using the Mendelian Randomization R package^22^. Analyses were stratified by sex, and association estimates represent the change in log(LV mass) per unit change in the phenotype corresponding to the GRS. An IVWA association p < 0.05 was considered significant. For T2D, a GRS was also constructed that excluded SNPs associated with BMI at p < 0.05.

## Supplementary information

Supplementary Information.

## Data Availability

G.W.A.S. summary statistics for LV Mass will be made available through dbGaP or can be obtained from the corresponding author. eMERGE data are available through dbGaP (phs000360.v3.p1).
