# Supplementary material for: The polygenic architecture of left ventricular mass mirrors the clinical epidemiology
Source: Sci Rep. 2020 May 5;10:7561. doi: 10.1038/s41598-020-64525-z (PMC7200691; doi:10.1038/s41598-020-64525-z)
Supplement: Supplementary file 1 — Supplementary Information. [file 41598_2020_64525_MOESM1_ESM.pdf]

The polygenic architecture of left ventricular mass mirrors the clinical epidemiology

Supplementary Materials

Jonathan D. Mosley, Rebecca T. Levinson, Eric Farber-Eger, Todd L. Edwards, Jacklyn N. Hellwege, Adriana M. Hung, Ayush Giri, Megan M. Shuey, Christian M. Shaffer, Mingjian Shi, Evan L. Brittain, Wendy K. Chung, Iftikhar J. Kullo, Adelaide M. Arruda-Olson, Gail P. Jarvik, Eric B. Larson, David R. Crosslin, Marc S. Williams, Ken M. Borthwick, Hakon Hakonarson, Joshua C. Denny, Thomas J. Wang, Charles M. Stein, Dan M. Roden and Quinn S. Wells

Supplementary figure 1

A

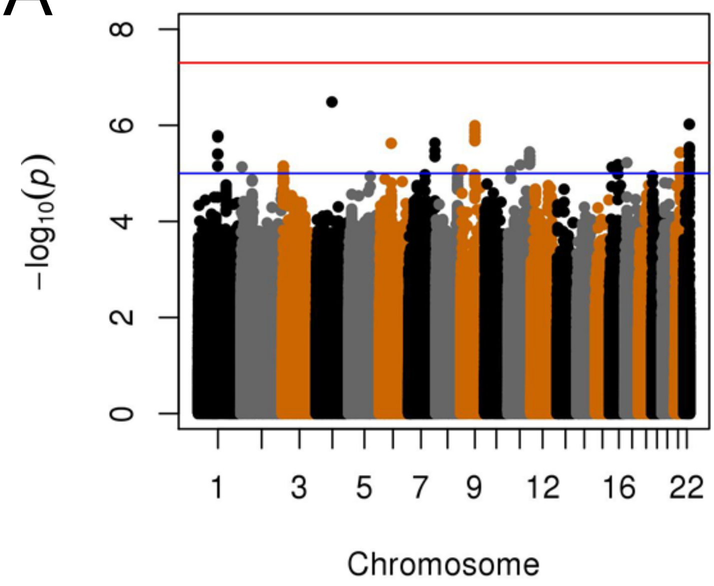

B

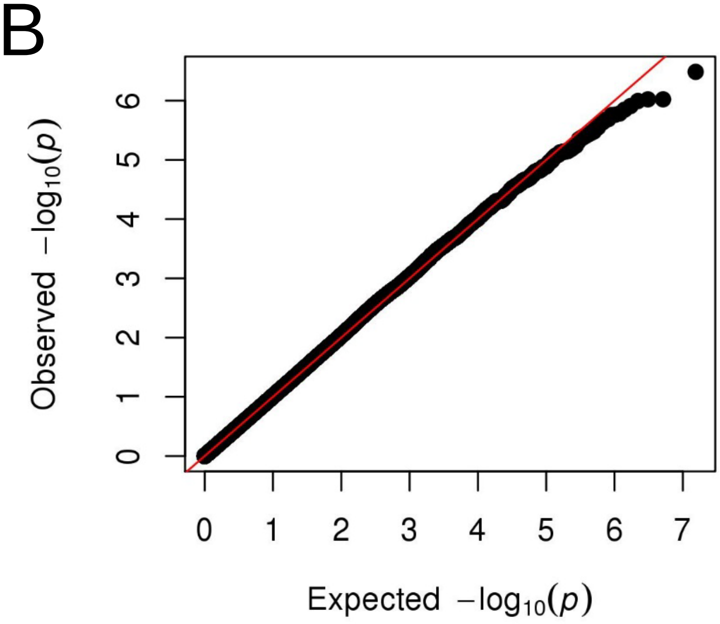

Supplementary Figure 1: Results of a GWAS analysis of LV mass. Results are based on an additive model adjusted for age, sex and 10 PCs. The figures show the (A) Manhattan plot and (B) the QQ plot..

Supplementary Table 1. Characteristics of the BioVU subjects with TTE measurements. Shown are the 100 most prevalent PheWAS diagnoses.

Characteristic	
Males [n (%)]	4,025 (53.0%)
Females [n (%)]	3,576 (47.0%)
Age at echo (years) [mean (s.d)]	64 (12)

Most common prevalent diagnoses:

	All Subjects		Males		Female	
**Diagnosis**	**Count**	**Prevalence**	**Count**	**Prevalence**	**Count**	**Prevalence**
Hypertension	6121	80.5%	3347	83.2%	2774	77.6%
Essential hypertension	6018	79.2%	3281	81.5%	2737	76.5%
Other symptoms of respiratory system	5702	75.0%	3037	75.5%	2665	74.5%
Cardiac dysrhythmias	5171	68.0%	2893	71.9%	2278	63.7%
Disorders of lipoid metabolism	5123	67.4%	2885	71.7%	2238	62.6%
Hyperlipidemia	5114	67.3%	2880	71.6%	2234	62.5%
Nonspecific chest pain	4647	61.1%	2466	61.3%	2181	61.0%
Shortness of breath	4413	58.1%	2361	58.7%	2052	57.4%
Ischemic Heart Disease	4359	57.3%	2684	66.7%	1675	46.8%
Fluid, electrolyte, & acid-base balance disorders	3965	52.2%	2156	53.6%	1809	50.6%
Coronary atherosclerosis	3929	51.7%	2501	62.1%	1428	39.9%
Malaise and fatigue	3818	50.2%	1879	46.7%	1939	54.2%
Other anemias	3686	48.5%	2005	49.8%	1681	47.0%
Pulmonary collapse; interstitial/compensatory emphysema	3659	48.1%	2148	53.4%	1511	42.3%
Other dyspnea	3422	45.0%	1865	46.3%	1557	43.5%
Electrolyte imbalance	3263	42.9%	1775	44.1%	1488	41.6%
Congestive heart failure, nonhypertensive	3205	42.2%	1853	46.0%	1352	37.8%
Diseases of esophagus	3164	41.6%	1602	39.8%	1562	43.7%
Pain in joint	3011	39.6%	1371	34.1%	1640	45.9%
Heart valve disorders	2976	39.2%	1678	41.7%	1298	36.3%
Cardiomegaly	2926	38.5%	1776	44.1%	1150	32.2%
Esophagitis, GERD & related diseases	2865	37.7%	1436	35.7%	1429	40.0%
Abdominal pain	2748	36.2%	1336	33.2%	1412	39.5%
GERD	2724	35.8%	1340	33.3%	1384	38.7%
Diabetes mellitus	2720	35.8%	1605	39.9%	1115	31.2%
Renal failure	2718	35.8%	1658	41.2%	1060	29.6%
Tobacco use disorder	2701	35.5%	1686	41.9%	1015	28.4%
Pleurisy	2700	35.5%	1556	38.7%	1144	32.0%
Congestive heart failure NOS	2684	35.3%	1552	38.6%	1132	31.7%
Atrial fibrillation & flutter	2678	35.2%	1643	40.8%	1035	28.9%
Hypertensive heart and/or renal disease	2654	34.9%	1544	38.4%	1110	31.0%
Cardiac conduction disorders	2645	34.8%	1610	40.0%	1035	28.9%
Type 2 diabetes	2641	34.7%	1560	38.8%	1081	30.2%
Atrial fibrillation	2614	34.4%	1602	39.8%	1012	28.3%
Cerebrovascular disease	2564	33.7%	1394	34.6%	1170	32.7%
Mixed hyperlipidemia	2528	33.3%	1450	36.0%	1078	30.1%
Cough	2442	32.1%	1209	30.0%	1233	34.5%
Hypotension	2398	31.5%	1417	35.2%	981	27.4%
Respiratory failure; insufficiency; arrest	2354	31.0%	1334	33.1%	1020	28.5%

Back pain	2331	30.7%	1070	26.6%	1261	35.3%
Osteoarthrosis	2321	30.5%	1061	26.4%	1260	35.2%
Edema	2225	29.3%	1086	27.0%	1139	31.9%
Mood disorders	2164	28.5%	967	24.0%	1197	33.5%
Symptoms/disorders of the urinary system	2152	28.3%	1033	25.7%	1119	31.3%
Other forms of chronic heart disease	2106	27.7%	1287	32.0%	819	22.9%
Other diseases of lung	2104	27.7%	1165	28.9%	939	26.3%
Overweight	2094	27.5%	1058	26.3%	1036	29.0%
Acute renal failure	2092	27.5%	1270	31.6%	822	23.0%
Tachycardia NOS	2084	27.4%	1116	27.7%	968	27.1%
Nausea and vomiting	2072	27.3%	930	23.1%	1142	31.9%
Urinary tract infection	2056	27.0%	748	18.6%	1308	36.6%
Hypopotassemia	2051	27.0%	1046	26.0%	1005	28.1%
Chronic ischemic heart disease	2049	27.0%	1435	35.7%	614	17.2%
Nonrheumatic mitral valve disorders	2037	26.8%	1117	27.8%	920	25.7%
Osteoporosis, osteopenia, & pathological fractures	2018	26.5%	693	17.2%	1325	37.1%
Hypotension NOS	2008	26.4%	1200	29.8%	808	22.6%
Hypovolemia	2005	26.4%	1100	27.3%	905	25.3%
Abnormal glucose	1999	26.3%	1147	28.5%	852	23.8%
Depression	1990	26.2%	870	21.6%	1120	31.3%
pulmonary heart disease	1978	26.0%	1119	27.8%	859	24.0%
Palpitations	1950	25.7%	879	21.8%	1071	29.9%
Long-term use of anticoagulants	1937	25.5%	1137	28.2%	800	22.4%
Fever of unknown origin	1937	25.5%	1078	26.8%	859	24.0%
Myocardial infarction	1927	25.4%	1287	32.0%	640	17.9%
Cardiac arrhythmia NOS	1913	25.2%	1142	28.4%	771	21.6%
Neurological disorders	1912	25.2%	1029	25.6%	883	24.7%
Hypercholesterolemia	1843	24.2%	990	24.6%	853	23.9%
Other abnormal glucose	1842	24.2%	1072	26.6%	770	21.5%
Pneumonia	1842	24.2%	1013	25.2%	829	23.2%
Chronic airway obstruction	1826	24.0%	992	24.6%	834	23.3%
Hypertensive heart disease	1809	23.8%	1006	25.0%	803	22.5%
Obesity	1799	23.7%	922	22.9%	877	24.5%
Acute posthemorrhagic anemia	1780	23.4%	1032	25.6%	748	20.9%
Disorders of the kidney & ureters	1772	23.3%	1087	27.0%	685	19.2%
Anxiety, phobic & dissociative disorders	1753	23.1%	720	17.9%	1033	28.9%
Other specified cardiac dysrhythmias	1736	22.8%	1009	25.1%	727	20.3%
Chronic renal failure	1729	22.7%	1087	27.0%	642	18.0%
Hypothyroidism	1717	22.6%	654	16.2%	1063	29.7%
Osteoarthrosis NOS	1711	22.5%	781	19.4%	930	26.0%
Ill-defined descriptions and complications of heart disease	1708	22.5%	972	24.1%	736	20.6%
Occlusion and stenosis of precerebral arteries	1661	21.9%	957	23.8%	704	19.7%
Diseases of white blood cells	1651	21.7%	902	22.4%	749	20.9%
Atherosclerosis	1642	21.6%	956	23.8%	686	19.2%
Hypothyroidism NOS	1630	21.4%	614	15.3%	1016	28.4%
Nonrheumatic aortic valve disorders	1606	21.1%	942	23.4%	664	18.6%
Cardiomyopathy	1593	21.0%	1049	26.1%	544	15.2%
Encounter for long-term use of anticoagulants/antithrombotics	1587	20.9%	974	24.2%	613	17.1%
Light-headedness and vertigo	1585	20.9%	744	18.5%	841	23.5%
Systolic or combined heart failure	1584	20.8%	1018	25.3%	566	15.8%
Syncope and collapse	1558	20.5%	846	21.0%	712	19.9%
Respiratory failure	1537	20.2%	835	20.7%	702	19.6%
Anxiety disorder	1532	20.2%	619	15.4%	913	25.5%
Primary pulmonary hypertension	1532	20.2%	843	20.9%	689	19.3%

Sleep disorders	1530	20.1%	719	17.9%	811	22.7%
Diverticulosis and diverticulitis	1516	19.9%	832	20.7%	684	19.1%
Chronic rheumatic disease of the heart valves	1501	19.7%	801	19.9%	700	19.6%
Diverticulosis	1452	19.1%	811	20.1%	641	17.9%
Encounter for long-term use of aspirin	1440	18.9%	890	22.1%	550	15.4%
Symptoms and disorders of the joints	1434	18.9%	662	16.4%	772	21.6%
Primary/intrinsic cardiomyopathies	1426	18.8%	944	23.5%	482	13.5%

Supplementary Table 2. Characteristics of the eMERGE and additional BioVU subjects. Shown are the 100 most common PheWAS diagnoses.

eMERGE subjects

Characteristic	
Males [n (%)]	14,268 (44.9%)
Females [n (%)]	17,503 (55.1%)
Age at echo [mean (s.d)]	1945 (1935-1955)

Most Common diagnoses

Diagnosis	Count	Prevalence
Hypertension	19753	62.2%
Disorders of lipoid metabolism	19751	62.2%
Hyperlipidemia	19668	61.9%
Essential hypertension	19433	61.2%
Other symptoms of respiratory system	18083	56.9%
Pain in joint	17994	56.6%
Cardiac dysrhythmias	14636	46.1%
Nonspecific chest pain	14382	45.3%
Osteoarthrosis	13963	43.9%
Back pain	13598	42.8%
Abdominal pain	13330	42.0%
Malaise and fatigue	12643	39.8%
Cough	11941	37.6%
Diseases of esophagus	11801	37.1%
Hypercholesterolemia	11652	36.7%
Osteoarthrosis NOS	11603	36.5%
Peripheral enthesopathies	11555	36.4%
Esophagitis, GERD & related diseases	11334	35.7%
Ischemic Heart Disease	11104	34.9%
Symptoms/disorders of the urinary system	10936	34.4%

BioVU subjects (excluding TTE subjects)

Characteristic	
Males [n (%)]	12,011 (44.4%)
Females [n (%)]	15,054 (55.6%)
Age at echo [mean (s.d)]	51.0 (20.2)

Most Common diagnoses

Diagnosis	Count	Prevalence
Hypertension	13185	48.7%
Essential hypertension	12958	47.9%
Other symptoms of respiratory system	12838	47.4%
Malaise and fatigue	11085	41.0%
Pain in joint	10365	38.3%
Disorders of lipoid metabolism	10305	38.1%
Hyperlipidemia	10264	37.9%
Nonspecific chest pain	10122	37.4%
Abdominal pain	9856	36.4%
Cardiac dysrhythmias	9548	35.3%
Diseases of esophagus	8392	31.0%
Fluid, electrolyte, & acid-base balance disorders	8354	30.9%
Esophagitis, GERD & related diseases	7843	29.0%
Other anemias	7801	28.8%
Shortness of breath	7674	28.4%
Back pain	7354	27.2%
GERD	7249	26.8%
Cough	6967	25.7%
Ischemic Heart Disease	6723	24.8%
Symptoms/disorders of the urinary system	6314	23.3%

Degenerative skin conditions and other dermatoses	10877	34.2%
Overweight	10783	33.9%
Other anemias	10772	33.9%
Acute upper respiratory infections	10763	33.9%
Other dyspnea	10637	33.5%
GERD	10357	32.6%
Cataract	10066	31.7%
Osteoporosis, osteopenia, & pathological fractures	10007	31.5%
Coronary atherosclerosis	9640	30.3%
Disorders of refraction and accommodation	9595	30.2%
Fluid, electrolyte, & acid-base balance disorders	9400	29.6%
Atopic or contact dermatitis	9211	29.0%
Shortness of breath	9152	28.8%
Urinary tract infection	8802	27.7%
Seborrheic keratosis	8729	27.5%
Obesity	8697	27.4%
Cardiac conduction disorders	8664	27.3%
Benign neoplasm of skin	8590	27.0%
Light-headedness and vertigo	8443	26.6%
Senile cataract	8355	26.3%
Mood disorders	8275	26.0%
Anxiety, phobic & dissociative disorders	8104	25.5%
Disorder of skin and subcutaneous tissue NOS	7961	25.1%
Benign neoplasm of colon	7942	25.0%
Depression	7920	24.9%
Diabetes mellitus	7787	24.5%
Spondylosis and allied disorders	7714	24.3%
Intervertebral disc disorders	7685	24.2%
Hearing loss	7675	24.2%
Type 2 diabetes	7627	24.0%

Nausea and vomiting	6314	23.3%
Mood disorders	6064	22.4%
Electrolyte imbalance	6049	22.3%
Osteoarthrosis	5979	22.1%
Coronary atherosclerosis	5932	21.9%
Other dyspnea	5876	21.7%
Urinary tract infection	5764	21.3%
Pulmonary collapse; interstitial/compensatory emphysema	5688	21.0%
Tobacco use disorder	5638	20.8%
Fever of unknown origin	5543	20.5%
Depression	5506	20.3%
Acute upper respiratory infections	5416	20.0%
Overweight	5368	19.8%
Anxiety, phobic & dissociative disorders	5330	19.7%
Diabetes mellitus	5250	19.4%
Mixed hyperlipidemia	5168	19.1%
Other headache syndromes	5051	18.7%
Osteoporosis, osteopenia, & pathological fractures	5040	18.6%
Type 2 diabetes	5001	18.5%
Hypovolemia	4646	17.2%
Hypercholesterolemia	4475	16.5%
Allergic rhinitis	4467	16.5%
Anxiety disorder	4464	16.5%
Edema	4337	16.0%
Hypertensive heart and/or renal disease	4329	16.0%
Renal failure	4295	15.9%
Peripheral enthesopathies	4218	15.6%
Hypothyroidism	4206	15.5%
Cerebrovascular disease	4200	15.5%
Obesity	4199	15.5%

Symptoms and disorders of the joints	7605	23.9%
Other headache syndromes	7567	23.8%
Diverticulosis and diverticulitis	7499	23.6%
Osteoarthritis; localized	7480	23.5%
Spondylosis without myelopathy	7463	23.5%
Superficial cellulitis & abscess	7447	23.4%
Other hypertrophic and atrophic conditions of skin	7407	23.3%
Sleep disorders	7328	23.1%
Osteopenia	7321	23.0%
Cerebrovascular disease	7309	23.0%
Heart valve disorders	7276	22.9%
Sprains and strains	7224	22.7%
Electrolyte imbalance	7179	22.6%
Disorders of synovium, tendon, and bursa	7172	22.6%
Edema	7120	22.4%
Actinic keratosis	7100	22.3%
Diverticulosis	7093	22.3%
Diseases of sebaceous glands	7073	22.3%
Renal failure	7069	22.2%
Enthesopathy	7052	22.2%
Hemorrhoids	7023	22.1%
Menopausal & postmenopausal disorders	6935	21.8%
Cervicalgia	6902	21.7%
Abdominal hernia	6881	21.7%
Nausea and vomiting	6698	21.1%
Anxiety disorder	6669	21.0%
Contusion	6534	20.6%
Hypothyroidism	6340	20.0%
Abnormal glucose	6292	19.8%
Abnormal findings on mammogram or breast exam	6273	19.7%
Tobacco use disorder	6271	19.7%
Hypothyroidism NOS	6141	19.3%
Other specified cardiac dysrhythmias	6141	19.3%

Osteoarthrosis NOS	4177	15.4%
Sleep disorders	4047	15.0%
Vitamin deficiency	3969	14.7%
Symptoms and disorders of the joints	3944	14.6%
Tachycardia NOS	3924	14.5%
Hypopotassemia	3913	14.5%
Hypothyroidism NOS	3893	14.4%
Pleurisy	3861	14.3%
Congestive heart failure, nonhypertensive	3842	14.2%
Pneumonia	3788	14.0%
Neurological disorders	3779	14.0%
Light-headedness and vertigo	3718	13.7%
Other diseases of lung	3712	13.7%
Superficial cellulitis & abscess	3707	13.7%
Palpitations	3696	13.7%
Cardiac conduction disorders	3663	13.5%
Cervicalgia	3613	13.3%
Acute sinusitis	3583	13.2%
Rash and other nonspecific skin eruption	3551	13.1%
Hypotension	3541	13.1%
Intervertebral disc disorders	3530	13.0%
Degenerative skin conditions and other dermatoses	3500	12.9%
Benign neoplasm of colon	3483	12.9%
Abdominal hernia	3450	12.7%
Heart valve disorders	3449	12.7%
Diverticulosis and diverticulitis	3445	12.7%
Disorders of the kidney & ureters	3428	12.7%
Atrial fibrillation & flutter	3387	12.5%
Vitamin D deficiency	3360	12.4%
Respiratory failure; insufficiency; arrest	3312	12.2%
Diseases of white blood cells	3305	12.2%
Diverticulosis	3294	12.2%
Atrial fibrillation	3289	12.2%

Other peripheral nerve disorders	6127	19.3%
Degeneration of intervertebral disc	6116	19.2%
Gastrointestinal hemorrhage	6107	19.2%
Pneumonia	6077	19.1%
Disturbance of skin sensation	6077	19.1%
Cardiac arrhythmia NOS	5961	18.8%
Ill-defined descriptions and complications of heart disease	5950	18.7%
Other abnormal blood chemistry	5882	18.5%
Dermatophytosis / Dermatomycosis	5870	18.5%
Fever of unknown origin	5865	18.5%
Atrial fibrillation & flutter	5853	18.4%
Allergic rhinitis	5849	18.4%
ngestive heart failure, nonhypertens	5841	18.4%
Astigmatism	5784	18.2%
Atrial fibrillation	5708	18.0%
Frequency of urination and polyuria	5706	18.0%
Abnormal movement	5687	17.9%

Gastrointestinal hemorrhage	3287	12.1%
Hearing loss	3258	12.0%
Acute pain	3240	12.0%
Cardiomegaly	3202	11.8%
Dysphagia	3173	11.7%
Abnormal glucose	3138	11.6%
Other symptoms involving abdomen and pelvis	3137	11.6%
Chronic airway obstruction	3134	11.6%
Skin neoplasm of uncertain behavior	3126	11.5%
Hypertensive heart disease	3106	11.5%
Osteoarthritis; localized	3095	11.4%
Disturbance of skin sensation	3092	11.4%
Congestive heart failure NOS	3027	11.2%
Spondylosis and allied disorders	3023	11.2%
Other disorders of intestine	3009	11.1%
Menopausal & postmenopausal disorders	3007	11.1%
Osteoporosis, NOS or other	3001	11.1%

Supplementary Table 3: PheWAS phenotypes associated LV mass at FDR q<0.1.

PheCode	Phenotype	Cases	Controls	Odds-ratio	95% CI	p-value	FDR p-value
278	Overweight	10282	37475	1.08	(1.05-1.11)	3.5E-08	2.5E-05
278.1	Obesity	8250	36493	1.09	(1.05-1.12)	5.6E-08	2.5E-05
278.11	Morbid obesity	3548	36319	1.10	(1.06-1.15)	7.9E-06	2.0E-03
401.1	Essential hypertension	22662	20492	1.06	(1.03-1.09)	1.0E-05	2.0E-03
425.1	Primary/intrinsic cardiomyopathies	2231	46830	1.13	(1.07-1.19)	1.2E-05	2.0E-03
401	Hypertension	24010	20492	1.06	(1.03-1.08)	1.6E-05	2.0E-03
411.4	Coronary atherosclerosis	11882	29867	1.06	(1.03-1.09)	2.0E-05	2.0E-03
411	Ischemic Heart Disease	13796	32202	1.06	(1.03-1.09)	2.1E-05	2.0E-03
425	Cardiomyopathy	2569	46841	1.11	(1.06-1.17)	2.3E-05	2.0E-03
428.1	Congestive heart failure NOS	5085	37458	1.09	(1.05-1.13)	2.3E-05	2.0E-03
428	Congestive heart failure, nonhypertensive	6682	37458	1.08	(1.04-1.11)	2.5E-05	2.0E-03
411.2	Myocardial infarction	4632	31854	1.09	(1.04-1.13)	5.5E-05	4.1E-03
586	Disorders of the kidney & ureters	4448	37758	1.08	(1.04-1.12)	7.3E-05	4.9E-03
250.2	Type 2 diabetes	9040	33956	1.06	(1.03-1.09)	7.6E-05	4.9E-03
250.3	Insulin pump user	1324	35092	1.14	(1.07-1.22)	9.8E-05	5.9E-03
428.3	Systolic or combined heart failure	2013	37458	1.12	(1.06-1.18)	1.1E-04	6.0E-03
585.1	Acute renal failure	3577	37758	1.09	(1.04-1.13)	1.4E-04	6.6E-03
415	pulmonary heart disease	3324	42094	1.09	(1.04-1.14)	1.4E-04	6.6E-03
586.2	Cyst of kidney, acquired	996	36665	1.16	(1.07-1.25)	1.4E-04	6.6E-03
250	Diabetes mellitus	9384	33956	1.06	(1.03-1.09)	1.6E-04	7.2E-03
416	Cardiomegaly	3710	40939	1.08	(1.04-1.13)	1.8E-04	7.7E-03
286.2	Long-term use of anticoagulants	4440	39127	1.08	(1.03-1.12)	3.0E-04	0.01
427.22	Atrial flutter	1395	25110	1.13	(1.06-1.21)	3.6E-04	0.01
427.2	Atrial fibrillation & flutter	6393	24628	1.07	(1.03-1.11)	4.3E-04	0.02
585.32	End stage renal disease	1168	37754	1.14	(1.06-1.22)	4.8E-04	0.02
411.1	Unstable angina (intermediate coronary syndrome)	2145	29301	1.10	(1.04-1.16)	7.9E-04	0.03
411.8	Chronic ischemic heart disease	3826	29859	1.08	(1.03-1.12)	1.3E-03	0.04
426	Cardiac conduction disorders	6840	26928	1.06	(1.02-1.10)	1.4E-03	0.04

427.21	Atrial fibrillation	5771	24292	1.06	(1.02-1.11)	1.4E-03	0.04
585.3	Chronic renal failure	5881	37767	1.06	(1.02-1.10)	1.7E-03	0.05
585	Renal failure	8339	37767	1.05	(1.02-1.08)	1.8E-03	0.05
428.2	Heart failure NOS	1694	37458	1.10	(1.04-1.17)	2.1E-03	0.06
427.4	Cardiac arrest & ventricular fibrillation	488	26918	1.19	(1.06-1.33)	2.2E-03	0.06
426.9	Cardiac pacemaker/device in situ	2394	26928	1.09	(1.03-1.15)	2.2E-03	0.06
272	Disorders of lipoid metabolism	22026	22638	1.04	(1.01-1.06)	2.7E-03	0.07
272.1	Hyperlipidemia	21741	22638	1.04	(1.01-1.06)	2.7E-03	0.07
426.2	AV block	1672	25310	1.11	(1.04-1.18)	2.8E-03	0.07
426.32	Left bundle branch block	882	24282	1.14	(1.05-1.24)	2.9E-03	0.07
585.34	Chronic Kidney Disease, Stage IV	1063	37767	1.12	(1.04-1.21)	2.9E-03	0.07
250.1	Type 1 diabetes	1793	35092	1.09	(1.03-1.16)	3.4E-03	0.07
327.32	Obstructive sleep apnea	3170	36287	1.07	(1.02-1.12)	3.4E-03	0.07
285.21	Anemia in chronic kidney disease	831	33345	1.13	(1.04-1.23)	3.5E-03	0.08
415.2	Primary pulmonary hypertension	2199	40926	1.08	(1.03-1.14)	3.7E-03	0.08
426.3	Bundle branch block	1714	26918	1.10	(1.03-1.18)	3.7E-03	0.08
401.22	Hypertensive chronic kidney disease	2832	21532	1.08	(1.02-1.14)	4.0E-03	0.08
276	Fluid, electrolyte, & acid-base balance disorders	10865	36334	1.04	(1.01-1.07)	4.3E-03	0.08
426.91	Cardiac pacemaker in situ	1753	26928	1.10	(1.03-1.17)	4.4E-03	0.08
335	Multiple sclerosis	1082	39411	1.11	(1.03-1.19)	4.6E-03	0.09
250.22	Type 2 diabetic nephropathy	1403	31651	1.10	(1.03-1.17)	4.8E-03	0.09
433	Cerebrovascular disease	7646	40246	1.05	(1.01-1.08)	5.0E-03	0.09
252	Disorders of parathyroid gland	970	44476	1.12	(1.03-1.21)	5.1E-03	0.09
426.7	Abnormal electrocardiogram	2300	26915	1.09	(1.03-1.15)	5.5E-03	0.09

Supplementary Table 4: Validating the genetic risk scores.

GRS	Validation Phenotype	Beta (se)	p-value
Body mass index (BMI)	BMI	0.24 (0.01)	$1x10^{-100}$
Coronary artery disease (CAD)	CAD	0.115 (0.011)	$7.8x10^{-24}$
Type 2 diabetes (T2D)	T2D	0.205 (0.012)	$8.7x10^{-63}$
Systolic blood pressure (SBP)	SBP	0.079 (0.011)	$5.5x10^{-13}$

Million Veteran Program: Consortium Acknowledgement for Manuscripts

MVP Executive Committee

- Co-Chair: J. Michael Gaziano, M.D., M.P.H.
- Co-Chair: Rachel Ramoni, D.M.D., Sc.D.
- Jim Breeling, M.D. (ex-officio)
- Kyong-Mi Chang, M.D.
- Grant Huang, Ph.D.
- Sumitra Muralidhar, Ph.D.
- Christopher J. O'Donnell, M.D., M.P.H.
- Philip S. Tsao, Ph.D.

MVP Program Office

- Sumitra Muralidhar, Ph.D.
- Jennifer Moser, Ph.D.

MVP Recruitment/Enrollment

- Recruitment/Enrollment Director/Deputy Director, Boston – Stacey B. Whitbourne, Ph.D.; Jessica V. Brewer, M.P.H.
- MVP Coordinating Centers
    - Clinical Epidemiology Research Center (CERC), West Haven – John Concato, M.D., M.P.H.
    - Cooperative Studies Program Clinical Research Pharmacy Coordinating Center, Albuquerque - Stuart Warren, J.D., Pharm D.; Dean P. Argyres, M.S.
    - Genomics Coordinating Center, Palo Alto – Philip S. Tsao, Ph.D.
    - Massachusetts Veterans Epidemiology Research Information Center (MAVERIC), Boston - J. Michael Gaziano, M.D., M.P.H.
    - MVP Information Center, Canandaigua – Brady Stephens, M.S.
- Core Biorepository, Boston – Mary T. Brophy M.D., M.P.H.; Donald E. Humphries, Ph.D.
- MVP Informatics, Boston – Nhan Do, M.D.; Shahpoor Shayan
- Data Operations/Analytics, Boston – Xuan-Mai T. Nguyen, Ph.D.

MVP Science

- Genomics - Christopher J. O'Donnell, M.D., M.P.H.; Saiju Pyarajan Ph.D.; Philip S. Tsao, Ph.D.
- Phenomics - Kelly Cho, M.P.H, Ph.D.
- Data and Computational Sciences – Saiju Pyarajan, Ph.D.
- Statistical Genetics – Elizabeth Hauser, Ph.D.; Yan Sun, Ph.D.; Hongyu Zhao, Ph.D.

MVP Local Site Investigators

- Atlanta VA Medical Center (Peter Wilson)
- Bay Pines VA Healthcare System (Rachel McArdle)
- Birmingham VA Medical Center (Louis Dellitalia)
- Cincinnati VA Medical Center (John Harley)
- Clement J. Zablocki VA Medical Center (Jeffrey Whittle)
- Durham VA Medical Center (Jean Beckham)
- Edith Nourse Rogers Memorial Veterans Hospital (John Wells)
- Edward Hines, Jr. VA Medical Center (Salvador Gutierrez)
- Fayetteville VA Medical Center (Gretchen Gibson)
- VA Health Care Upstate New York (Laurence Kaminsky)
- New Mexico VA Health Care System (Gerardo Villareal)
- VA Boston Healthcare System (Scott Kinlay)
- VA Western New York Healthcare System (Junzhe Xu)
- Ralph H. Johnson VA Medical Center (Mark Hamner)
- Wm. Jennings Bryan Dorn VA Medical Center (Kathlyn Sue Haddock)
- VA North Texas Health Care System (Sujata Bhushan)
- Hampton VA Medical Center (Pran Iruvanti)
- Hunter Holmes McGuire VA Medical Center (Michael Godschalk)
- Iowa City VA Health Care System (Zuhair Ballas)
- Jack C. Montgomery VA Medical Center (Malcolm Buford)
- James A. Haley Veterans' Hospital (Stephen Mastorides)
- Louisville VA Medical Center (Jon Klein)
- Manchester VA Medical Center (Nora Ratcliffe)
- Miami VA Health Care System (Hermes Florez)
- Michael E. DeBakey VA Medical Center (Alan Swann)
- Minneapolis VA Health Care System (Maureen Murdoch)
- N. FL/S. GA Veterans Health System (Peruvemba Sriram)
- Northport VA Medical Center (Shing Shing Yeh)
- Overton Brooks VA Medical Center (Ronald Washburn)
- Philadelphia VA Medical Center (Darshana Jhala)
- Phoenix VA Health Care System (Samuel Aguayo)
- Portland VA Medical Center (David Cohen)
- Providence VA Medical Center (Satish Sharma)
- Richard Roudebush VA Medical Center (John Callaghan)
- Salem VA Medical Center (Kris Ann Oursler)
- San Francisco VA Health Care System (Mary Whooley)
- South Texas Veterans Health Care System (Sunil Ahuja)
- Southeast Louisiana Veterans Health Care System (Amparo Gutierrez)

- Southern Arizona VA Health Care System (Ronald Schifman)
- Sioux Falls VA Health Care System (Jennifer Greco)
- St. Louis VA Health Care System (Michael Rauchman)
- Syracuse VA Medical Center (Richard Servatius)
- VA Eastern Kansas Health Care System (Mary Oehlert)
- VA Greater Los Angeles Health Care System (Agnes Wallbom)
- VA Loma Linda Healthcare System (Ronald Fernando)
- VA Long Beach Healthcare System (Timothy Morgan)
- VA Maine Healthcare System (Todd Stapley)
- VA New York Harbor Healthcare System (Scott Sherman)
- VA Pacific Islands Health Care System (Gwenevere Anderson)
- VA Palo Alto Health Care System (Philip Tsao)
- VA Pittsburgh Health Care System (Elif Sonel)
- VA Puget Sound Health Care System (Edward Boyko)
- VA Salt Lake City Health Care System (Laurence Meyer)
- VA San Diego Healthcare System (Samir Gupta)
- VA Southern Nevada Healthcare System (Joseph Fayad)
- VA Tennessee Valley Healthcare System (Adriana Hung)
- Washington DC VA Medical Center (Jack Lichy)
- W.G. (Bill) Hefner VA Medical Center (Robin Hurley)
- White River Junction VA Medical Center (Brooks Robey)
- William S. Middleton Memorial Veterans Hospital (Robert Striker)
